# Evaluating a Mobile App Supporting Evidence-Based Parenting Skills: Thematic Analysis of Parent Experience

**DOI:** 10.2196/53907

**Published:** 2024-09-05

**Authors:** Nathan Hodson, Peter Woods, Juan Luque Solano, Charlotte Talbot, Domenico Giacco

**Affiliations:** 1 Price School of Public Policy, University of Southern California, 650 Childs Way, Los Angeles, CA, 90089, United States, 1 2133480086; 2 Warwick Medical School, University of Warwick, Coventry, United Kingdom; 3 University of Buckingham, Milton Keynes, United Kingdom; 4 Birmingham Medical School, Birmingham, United Kingdom

**Keywords:** digital microintervention, parenting app, product management, parent, parents, parenting, app, apps, usability, acceptability, family, families, interview, interviews, pediatric, pediatrics, child, children, youths, experience, experiences, attitude, attitudes, opinion, perception, perceptions, perspective, perspectives, acceptance, behavior, behaviors, disruptive behavior, thematic analysis

## Abstract

**Background:**

Disruptive behavior disorders are among the most common disorders of childhood, and evidence-based parenting programs are the first-line treatment. Digital microinterventions have been proposed as one possible means of supporting parenting style change by giving parents in-the-moment advice about how to respond to challenging behavior. Until now, no digital microintervention supporting evidence-based parenting skills programs has been evaluated.

**Objective:**

The aim of this study is to evaluate the subjective experience of parents using a digital microintervention to support evidence-based parenting skills, with particular attention to acceptability, usability, family relationships, and parents’ values.

**Methods:**

We conducted serial interviews with 11 parents of 33 children before and after spending 3 weeks using an app including 3 digital microinterventions. Parents were recruited via local authorities in the Midlands region of the United Kingdom. Previous participation in a parenting program was an inclusion criterion. Interviews explored family composition; child behavior problems; and experience of using the mobile app, including barriers to use. Thematic analysis was conducted from a user-centered design perspective, and illustrative case vignettes were produced.

**Results:**

Many parents used the app in ways that helped them rather than strictly following the instructions they were given. Parents described a range of barriers to using the app including practical problems and failure to change child behavior. Parents and children responded in a variety of ways to the use of the phone, with many wholeheartedly embracing the convenience of technology. Case vignettes illustrate the uniqueness of each family’s experience.

**Conclusions:**

Parents’ use of a mobile app supporting evidence-based parenting skills is difficult to predict due to the unique challenges each family encounters. Many parents found it an acceptable and helpful addition to family life, but increased personalization is likely to be key to supporting parents. Future digital microintervention developers should keep in mind that parents are likely to use the app pragmatically rather than following instructions, may struggle to use a complex app under pressure, and are likely to hold complex feelings about parenting with an app.

## Introduction

Disruptive behavior disorders such as conduct disorder and oppositional defiant disorder are among the most common mental health conditions of childhood [1]. Untreated children with conduct disorder persistently violate the rights and boundaries of other children as well as adults [2]. Although phenotypes vary, young people presenting with some of the criteria for conduct disorder are at high risk for future mental illness, educational failure, and contact with the criminal justice system [3-5].

This study evaluated an app that was designed to help parents to use evidence-based parenting skills (including praise, age-appropriate time-out, and relaxation exercises), which are shown to improve children’s behavior problems such as angry outbursts, violence, and refusing to follow instructions.

Evidence-based parenting skills programs are the first-line treatment for disruptive behavior disorders according to the National Institute for Health and Care Excellence [6]. Triple P, Incredible Years, and Chicago Parenting Program are supported by evidence from systematic reviews and meta-analyses. Changes in parenting style can lead to children building stronger self-esteem and learning to understand and respond to limits [7,8]. These programs can have long-term benefits; randomized controlled trials have shown that they set children on a path for better relationships, reduced criminal activity, and fewer behavioral diagnoses up to 8 years later [9].

Incredible Years, Triple P, and Chicago Parenting Program tend to draw on a range of didactic approaches to equip parents with new skills. The programs are frequently run as groups with 6‐12 parents meeting weekly for 3 or 4 months, during which time, they use roleplay, videos, and group discussions to learn new skills [10]. Programs generally begin by supporting parents to improve their relationship with their child through positive play and showing warmth before moving to equip parents with proportionate and effective approaches to setting limits such as ignoring unwanted behavior, distracting the child, and introducing natural or logical consequences.

This paper focuses on 3 particular skills. The first is praise: praising a child’s successes is an important part of building self-esteem, but vague praise of children with low self-esteem or, worse, backhanded praise, can damage the parent-child relationship [11]. The second is mindfulness: learning to calm raised emotions is a valuable skill for children with behavior problems, but there is a limited emotional window where children are responsive to mindfulness [12]. The third is a skill called “time-out” in Incredible Years and “quiet time” in Triple P, which provides an effective means of setting limits when carried out correctly. However, parents often struggle to use this skill, and resources available on the web conflict with the evidence for an effective time-out [13].

Changing parent behavior remains a significant challenge. One important consideration is that children’s behavior often begins to get worse as parents introduce evidence-based parenting techniques, so-called “extinction bursts,” which can wrongly give parents the impression that the techniques are not working [14]. A systematic review of face-to-face parenting programs for disruptive behavior found that only half of parents who start programs finish them [15].

The advice offered on apps and websites sometimes runs contrary to the evidence [13,16]. Although online programs provide an alternative to face-to-face programs, engagement appears to be even lower than in face-to-face programs, and evidence of lasting behavior change is weaker [17,18]. Extinction bursts are one reason for poor engagement: worsening behavior means parents lose faith in the program and quit. One parenting skills practitioner described to us the period between parental behavior change and child behavior improvement as the “giving-up gap.” At the same time, many parents fail to implement parenting techniques learned in a calm classroom once they are in a hectic home environment. This is in keeping with Loewenstein’s concept of the hot-cold empathy gap: in a calm state, people struggle to imagine the challenges they will face when they are emotionally aroused [19].

Given these 2 challenges, it is worthwhile considering whether more engaging digital interventions could change behavior. Baumel et al [20] called for digital microinterventions to help parents achieve change in parenting style and downstream improvements for parents. They define digital microintervention as “highly focused interventions delivered in the context of a person’s daily life with little burden on the individual” [20]. Parents report lacking free time and having limited cognitive bandwidth for learning new skills, and as such, immediate interventions with little burden have great potential [21]. Digital microinterventions have the potential to address the giving-up gap by helping parents notice their achievements and overcome the hot-cold empathy gap by giving guidance in the heat of the moment.

Despite Baumel et al’s [20] call to action, several unknowns remain. The cognitive constraints parents face may make an app overwhelming or may make prompts on an app particularly useful. Parents may prefer not to use a digital device to support parenting or may find it convenient. Parents may find that they prefer comprehensive instructions on parenting rather than a highly focused intervention. No digital microintervention has previously been used to guide parents through evidence-based skills in the moment when children are displaying disruptive behaviors.

In this paper, we aimed to evaluate the subjective experience of parents using a digital microintervention to support evidence-based parenting skills, with particular attention to acceptability, usability, family relationships, and parents’ values.

## Methods

### Context and Target Population

Participants were recruited through several parenting programs run by local authorities across the Midlands region of the United Kingdom. Recruitment was supported by parenting program practitioners who advertised the study by emailing the study invitation to parents who had recently completed a program or by directly inviting parents who attended their final sessions. Others were recruited by word of mouth around the local authority. A participant information leaflet was shared with interested parents. We aimed to establish a sample of 10 participants in keeping with other recent pilots of parenting apps and following the 10±2 rule in usability testing [22,23]. Included parents were aged 18 years and older and had a child aged 2‐10 years. Parents were included if they had sufficient concerns about their child’s behavior to be referred to a parenting skills program. By only including parents who had previously benefited from a parenting skills program, we ensured that they had received support with the basic components of parenting skills. The intervention is intended for use by parents who have attended a parenting skills program so this inclusion criterion was essential to the external validity of the intervention. Parents were excluded if they did not have an Android or iOS smartphone, which could use the app.

### Intervention

We previously developed a mobile app for iOS and Android through an iterative coproduction process involving 42 parenting skills practitioners reported elsewhere [24]. This app guided parents through using praise, mindfulness, and time-out or quiet time. It guided parents through using praise with a list of scrollable images with simple instructions on them. It guided parents through mindfulness by including a visual graphic helping children use “box breathing” as well as a guide walking parents through using a sensory grounding technique [25].

### Procedures

Each participant was interviewed twice in order to develop a rich understanding of how the digital microintervention fitted into family life [26]. At interview 1, participants completed a consent form, were offered a participant information form, and had the opportunity to ask any questions about the research. Then, they answered questions about their family composition, parenting style, and experience of parenting skills. They also talked about their child’s behavior problems and the strategies they use to manage these. Then, they were given a tour of the app and supported to download it to their own mobile device. They were asked about their initial responses to the app, including which features they thought could be useful or unhelpful. Topic guides are reported in Multimedia Appendix 1.

When they were shown the app, little specific psychoeducation was delivered, as they had already attended a parenting program. However, the app included suggestions to only use time-out or time in under circumstances of oppositional behavior or violence in keeping with best practice. Parents were encouraged to use the mindfulness activities with and without their children as they wished. They were encouraged to review the praise guidance, which offered very simple prompts to praise children frequently.

At the end of interview 1, participants received a US $32 shopping voucher and were invited to use the app and book a second interview about 3 weeks later following similar app pilots [27,28]. At interview 2, parents were asked whether they had used the app and how they had found the experience. Parents were prompted to talk about any features they had found helpful and any problems they had encountered. The whole incentive for participating was given during interview 1 to make it easier for parents to exit the study if they wished to.

All interviews were conducted remotely by NH and CT via Microsoft Teams or WhatsApp Voice Call and recorded with consent. All interviews were audio recorded and transcribed. If participants wished to have a friend or family member present during the interviews, this was accommodated. Transcripts were not returned to participants for comments.

### Analysis

Analysis was conducted by NH and JLS. Thematic analysis was conducted using NVivo (version 1.5; Lumivero) and followed the principles set out by Braun and Clarke [29,30] using an inductive approach with a theoretical underpinning in user-centered design. Analysis was also informed by a narrative lens in order to develop a richer understanding of how the digital microinterventions had interplayed with the dynamic stories of the families because “narratives provide us with access to people’s identity and personality” [31]. The narrative approach also helps present the uniqueness of each family’s story. Both NH and JLS read and reread the data before generating initial codes and searching for themes. Themes were reviewed discursively and named by NH. The coding framework is given in Table 1 and findings are discussed in detail below.

**Table 1.** Coding framework and illustrative quotations.

Themes		Representative quotations
**Theme 1: Unexpected uses of the app**		
	1.1: Different children	“I only tried it twice in my 10 year old cause I found he got more annoyed. I probably used it about five times with my 4 year old.”
	1.2: Learning the app	“I spent the next day like on the app, seeing what was on there and stuff”
	1.3: Different timers	“I’ve just remembered what it said and it’s like if it’s three minutes, or whatever, I’ve just put on my Google (dot)”
	1.4: Specific contexts	“I’ve used it generally every night, before she goes to bed, with my little one. We do a little bit of breathing to like, calm down”
**Theme 2: Not using the app**		
	2.1: Phone unavailable	“It’s been hard if my phones dying of battery”
	2.2: Forgetting	“I think it’s remembering in the moment to actually pick up my phone and think to use it when it’s happening.”
	2.3: Urgency	“I don’t know if you’re able to do voice activation or just an easier way to access those particular parts where you need them in that moment.”
	2.4: Not getting good results	“He got put in time out cause he’d hit me and he’d hit his younger brother. And I had to keep putting him back in time out, like because he kept going. And I kept making him sit. But like even after the time he just, he was just really upset.”
**Theme 3: Parenting with a mobile app**		
	3.1: Surprised by mobile parenting	“Before I knew about this, I never even considered there would be an app for this sort of thing”
	3.2: Using a phone is inappropriate	“I have issues as a professional with electronics and devices and as a parent and I can limit it, but in this case I don’t see it as a problem”
	3.3: Phones are helpful and convenient	“It’s not a distraction. it’s a useful tool to have”

Full transcripts include extensive personal content relating to children so they are not presented. Instead, anonymized brief narrative case studies illustrate the individuality of each included family. Each relates to a specific interviewee and draws together examples of their family life. Two brief case studies are presented in the *Results* section (see Multimedia Appendix 2 for others). The results were presented in keeping with the COREQ (Consolidated Criteria for Reporting Qualitative Research) [32].

### Ethical Considerations

Ethics approval was obtained from the Biomedical and Scientific Research Ethics Committee at the University of Warwick (BSREC 34/22‐23). Informed consent was obtained from all participants, and data were anonymized.

### Research Team and Reflexivity

NH and CT conducted the interviews, and participants were aware that NH was a psychiatrist with experience supporting parents concerned about their children’s behavior and well-being. Participants were referred to the research from a trusted source, the parenting team already supporting them, so participants may have expected the digital microintervention to be useful. NH and CT therefore actively sought to draw out the problems with the digital microintervention, but 2 participants nevertheless made apologetic comments including “I’m not saying that this is any reflection on the app at all” and “I looked through it like last night just kind of to refresh it. I wouldn’t say I look at it every, like every day.” When participants made comments along these lines, they were reminded that the aim of the research was to identify any problems, not just strengths.

The analysts who contributed to the thematic analysis and editing of the brief case studies, PW and JLS, had limited previous contact with these particular families but were involved in developing the app so they contributed a more technology-focused perspective than NH. DG was not directly involved in data collection or analysis or software development so he provided a different set of perspectives.

## Results

### Participants

#### Overview

In total, 11 parents of 33 children were included. All were female, and all had at least 1 child aged 2‐10 years. Among the participants’ 33 children, 1 was 18 years, and 6 others were 10 years and older. One was younger than 2 years. Only 1 was an only child. All used social media, most commonly Instagram, Facebook, and WhatsApp. Table 2 gives the characteristics of the included parents.

On average, interview 1 lasted 38 minutes and 42 seconds, and interview 2 lasted 21 minutes and 8 seconds. One participant did not attend a second interview and did not give a reason. All 10 parents who attended interview 2 reported that they had used the app in some form.

**Table 2.** Characteristics of included parents.

Topic	Parents (N=11), n (%)
Parents with more than 1 child	10 (91)
Any child with autistic spectrum disorder or attention-deficit/hyperactivity disorder	4 (36)
Use of time-out or naughty step or quiet corner	10 (91)
Past use of parenting app	2 (18)
Past use of parenting books	4 (36)
Use of social media	11 (100)
Lived with partner	7 (63)

#### Parenting Skills Background

Four children had diagnoses of attention-deficit/hyperactivity disorder or autistic spectrum disorder. Four participants were the only adults living at home. In total, 4 had used parenting books in the past, but only 2 had used a parenting app before. Only 1 had never used any variant of time-out or quiet time.

Participants reported a range of techniques or methods they took to managing behavioral and emotional problems prior to being offered the app. These included evidence-based techniques like distracting children, ignoring unwanted behavior, or using star charts. Some focused on managing emotions through calming breathing, coloring, or using an anger scale to notice when they are becoming upset. Other parents mentioned negative reinforcement through shouting, and one suggested other parents used corporal punishment. Encouraging physical touch was also identified as a useful emotion regulation technique, and predictable meal plans were important to another family. One parent mentioned using melatonin to manage behavior problems.

#### Comments Particular to This App

Reported strengths of the app including the mindfulness techniques and the advice on using praise, reducing excessive punishment, impulsivity, and inconsistency. They also approved of the prompts to remind parents to use positive parenting skills. They noted some weaknesses of the app including technical bugs, navigation difficulties, as well as the calming breathes being too slow for small children.

### Thematic Analysis: Parents’ Experience of Using a Parenting App

To address out main research question, we conducted a thematic analysis of the interviews in relation to parent’s experience of using the app. Table 1 shows the coding framework.

#### Theme 1: Unexpected Uses of the App

##### Theme 1.1: Different Children

Parents reported that they had used the app with some children but not with others. This is certainly appropriate for parents with older or younger children, but parents even found that not all of their children in the age range 2‐10 years had been responsive to the app. This ran contrary to our expectation that the app would increase consistency between children by supporting parents to use the same activities with all age-appropriate children. Several had tried the app with more than 1 child before deciding which one it was best suited to.

*I’ve tried it once with (child 1) because she doesn’t tend to get to a point where she needs it. Normally if I say to her “calm” it stops. It’s more for (child 2) I’ve used it for the calming time which has worked quite nicely and I think he’s liked it.*

[Participant 4, interview 2]

*I think I only tried it twice in my 10 year old cause I found he got more annoyed. I probably used it about five times with my 4 year old.*

[Participant 9, interview 2]

##### Theme 1.2: Learning the App

Although we expected the app to be used as a digital microintervention in the heat of the moment, some parents used it as a way of learning instead. Some read the information and memorized the approaches to the problems. Others used it like a game to “play through” the possible problems and prepare a strategy. One parent described it as “almost like, you know, pretending to click through to the options to see what was behind it” [Participant 11, interview 2].

*After our discussion, yeah, I spent the next day like on the app, seeing what was on there and stuff. And I was like. OK, that’s handy.*

[Participant 9, interview 2]

The praise section was particularly well suited to being used for learning only and then applying the skills at another time. Although the app included prompts and reminders to use praise, many parents primarily read through the praise advice.

*It’s like a slideshow of pictures and with some information and it’s regarding when to give children praise and there was a few other things on there, which I found was a really good read.*

[Participant 2, interview 2]

##### Theme 1.3: Different Timers

Although parents used the mindfulness and praise advice as recommended, they often took the time-out information but used different timers around the home instead of the app’s built-in timer. Once they had learned how the app worked, some preferred to link it to a timer that their child could see as well or a timer in a different space in the house.

*So I’ve just remembered what it said and it’s like if it’s three minutes, or whatever, I’ve just put on my Google and just been like “Ohh hey Google, set the timer for three minutes.”*

[Participant 6, interview 2]

*We have some egg timers and we have the ticking timers as well so I tend to use them anyway. So I wouldn’t necessarily use it on via the app. But if I was a parent who wasn’t already using the timers that I’ve got, then I think it was a good idea on the app.*

[Participant 2, interview 2]

##### Theme 1.4: Specific Contexts

Some parents reported that they primarily used the app in particular settings. The mindfulness section was intended for parents to use with their child in a relaxed state so they would be prepared to apply the same skills in a difficult context. This may have been due to a change in the environment or a daily routine.

*We’ve used it out and about we used it on holiday. We used it in the airport. We’ve used it like when we’ve been out in the car and things like that, and she’s getting really good at using it herself.*

[Participant 10, interview 2]

*I’ve used it generally every night, before she goes to bed, with my little one. We do a little bit of breathing to like, calm down and calm down function quick timer and she seems to like that.*

[Participant 5, interview 2]

### Theme 2: Not Using the App

#### Theme 2.1: Phone Unavailable

Some parents reported that they did not use the app because their phone was not in hand at the moment when they needed it. This was generally because it was somewhere else around the house, possibly on charge or being used by another child.

*Maybe one of the kids is watching YouTube on the phone or like kids tube or whatever, so I can’t just take it off one of the other children because one of them’s misbehaving.*

[Participant 6, interview 2]

*It’s been hard if my phones dying of battery and stuff like that.*

[Participant 9, interview 2]

#### Theme 2.2: Forgetting

Several parents reported forgetting to use the app. The app required a change of routine and parenting approach for them. Emotional and behavioral problems did not immediately trigger parents to pick up their phones.

*I think it’s remembering in the moment to actually pick up my phone and think to use it when it’s happening.*

[Participant 11, interview 2]

*I think I could use it, but I just think I've forgotten about it and I just need to get it in my head to use it as a strategy more in the house as well.*

[Participant 10, interview 2]

#### Theme 2.3: Urgency

Rather than interpreting their experience as “forgetting,” some parents reported that the situation had been too urgent for them to get their phones out. They described it as a rational and deliberate choice not to use the app in the heat of the moment. One parent suggested that a voice-controlled app would be easier to use when emotions are raised.

*If (child 1) is hitting (child 2), I wouldn’t necessarily think “I’ve got go and get my phone” or “I’m gonna have to look at my phone at the app”, I’d just be like try and think about like what I need to do or try to manage the situation.*

[Participant 6, interview 2]

*I don’t know if you’re able to do voice activation or just an easier way to access those particular parts where you need them in that moment.*

[Participant 2, interview 2]

#### Theme 2.4: Not Getting Good Results

Some parents did not use the time-out or quiet-time components because they did not achieve a change in behavior and because children were resistant. In some children, this was due to feeling that time-out was age-inappropriate, and in others, it was due to time-out being too difficult. There was no indication that children’s resistance was specifically linked to the app.

*He got put in time out cause he’d hit me and he’d hit his younger brother. And I had to keep putting him back in time out, like because he kept going. And I kept making him sit. But like even after the time he just, he was just really upset.*

[Participant 6, interview 2]

*When my son was in a real mood he’d pick (a chair) up and, you know, smash it against a door or something.*

[Participant 5, interview 2]

### Theme 3: Parenting With a Mobile App

#### Theme 3.1: Surprised by Mobile Parenting

Some parents were surprised that they were being offered an app to help with parenting. They reported that some of their children were surprised to find their parents using an app to guide evidence-based parenting rather than entertainment. Although initially surprised by the app, they reported rapidly becoming used to the phone and seeing it as an implement, which parents could use well or not.

*Before I knew about this, I never even considered there would be an app for this sort of thing.*

[Participant 3, interview 1]

*First time I got it out, he thought I was gonna let him watch YouTube. But no, no, this is something different.*

[Participant 9, interview 1]

*It’s a tool, isn’t it? You’re using it as a tool just the same you would as a as a piece of paper.*

[Participant 4, interview 2]

#### Theme 3.2: Using a Phone Is Inappropriate

Some parents were worried about using a phone as part of parenting. Only 1 parent strongly believed it was wrong, suggesting that a phone could form a barrier. Other parents expressed qualified concerns, which were overall outweighed.

*I also don’t think it’s good practise to be using your phone when you’re trying to sort that. If my children need attention and they’re needing intermediate intervention there and then, it’s not good for me to be having my phone in my hand.*

[Participant 2, interview 2]

*Some people don’t like to see phones and stuff right before bed, but it doesn’t seem to really affect her.*

[Participant 5, interview 2]

*I have issues as a professional with electronics and devices and as a parent and I can limit it, but in this case I don’t see it as a problem.*

[Participant 4, interview 2]

#### Theme 3.3: Phones Are Helpful and Convenient

Parents who compared the phone with other parenting resources could also identify advantages to using phones. The responsiveness and graphic interface of smartphones were seen as advantages. The possibility of a prompt card they could take anywhere was also seen as a strength. The use of the phone also created a warning sign, giving children a chance to de-escalate.

*There’s only so many visual aids I can have, you know? I mean, I know I printed out something that was like quite a nice little caption type stuff with the plan to laminate it, to put it in his room. But I’m like…actually in the heat of the moment…he doesn’t tend to go to sit in his room in his day.*

[Participant 11, interview 2]

*It’s not a distraction. it’s a useful tool to have, especially when you’re thinking “what should I do?” or “how should I do it?”*

[Participant 1, interview 2]

*I think even (child), when he’s watching me, he knows the phone comes out, he knows what’s coming next as well.*

[Participant 4, interview 2]

### Illustrative Case Vignettes

The case vignettes illustrated the way parents talked about their family life and the challenges they faced as well as capturing the personality of the parents and the way the relationships within their families impacted the response to the app. The vignettes draw attention to how siblings who display less prominent behavioral problems were sometimes able to use the app to regulate their emotions. The humor and closeness within families are highlighted by the way parents and children respond to the app. Textboxes 1 and 2 give illustrative examples of the case vignettes, and Multimedia Appendix 2 gives all 10 case vignettes.

Textbox 1. **Illustrative case vignette 1 (participant H).**

Before she had her twins - both 3 years old now - H used to work at a parenting assessment centre. She also has a 6 year old who can become violent and sometimes hits her. She finds it hard to know how to react. She’s completed Triple P in the past and he is awaiting a neurodevelopmental assessment. H was interested in trying out the app to give her new ideas and resources for managing this violence. She sometimes used time outs, but she found it difficult because their house is quite open plan so there’s not much privacy. More likely, her 6 year old announces his own time out and takes himself off to his bedroom.

She read through the information pages when she got the app and tried to start using more praise. She was surprised to see that she didn’t have to insist her children apologised when they were done and found that this change made life easier. She mentioned she had re-read the information pages before we met. In fact, she had memorised so much of the information on the app that she did not use the timer; instead she used the kitchen Alexa and the Google Home in the lounge to set the timers. The main problem with the app is that she doesn’t always have her phone on her; then again sometimes the smart speakers do not recognise her voice so no technology is perfect.

Textbox 2. **Illustrative case vignette 2 (participant V).**

Sometimes when your child is really winding you up and upsetting her siblings, V explains, it is hard to manage your own emotions. When everybody has fallen out, you know what they really need is a hug to make everything ok again, but you don’t really feel like hugging them. It’s good to have the app as a reminder to do that.

V has four children. It’s not that they’re naughty children but they do get really upset if she buys the wrong things from the shop. She tries to take their PS5 off them when they are badly behaved. She doesn’t smack her children but she thinks some people probably do and reckons that the app creates an alternative model for their relationship, even suggesting that ending with a hug will have positive oxytocin effects on family life.

She used the app 2‐3 times each week, including half term, but didn’t need it the week before we spoke. Her son got the hang of taking 30 seconds of calming down, but her daughter was a little confused about what she was supposed to do. By the end of the three weeks her son knew what was going to happen when she took her phone out and could anticipate calming time.

## Discussion

### Summary of Results

This study aimed to evaluate the subjective experience of parents using a digital microintervention to support evidence-based parenting skills. In total, 11 parents of 33 children were interviewed before and after using a mobile app hosting 3 digital microinterventions, and thematic analysis was performed. Many parents used the app in ways they found convenient rather than strictly following the instructions they were given. Several parents experienced barriers to using the app including not having the phone to hand, forgetting about the app, the situation being too urgent, or not finding they were getting good results. Parents and children responded differently to the use of the phone; some were surprised, others explained nuanced concerns, and others wholeheartedly embraced the convenience of mobile technology. Future digital microintervention developers should keep in mind that parents are likely to use the app pragmatically rather than following instructions, may struggle to use a complex app under pressure, and are likely to hold complex feelings about parenting with an app.

### Comparison With the Literature

The main problems parents noticed related to user interface problems including occasional freezing and navigation problems. Similar problems have been described in depth by MacKinnon et al [33], who described many practical challenges that arise in the implementation of an app for parents. As in their study, the app contained bugs due to the rapid build of the app under cost pressure. However, we suggest it is more efficient to run early tests of rapidly developed apps, which may contain bugs, in order to evaluate whether the underlying theory of behavior change works well rather than build a perfect app before testing it, in keeping with the principle of continuous innovation [34].

Many parents used the app in ways that were unexpected by researchers and out of keeping with the instructions they were given. Our intervention differed from common modes of parenting skills training, such as facilitator-led groups or e-learning videos, in that our intervention could be used in different ways [8,35]. We suggest digital microinterventions aiming to empower parents with digital tools are likely also to benefit from a group facilitator.

Parents held diverse views over whether using their phones to support their child’s behavioral and emotional well-being was appropriate, both in terms of whether they supported or opposed it and how far their views were nuanced or rigid. Previous research has revealed wide heterogeneity of attitudes throughout digital parenting, but there is little previous research on attitudes to using parenting apps, partly because the rapid pace of technological change precludes the formation of social norms [36]. Given the complex interaction of difficult family circumstances and shifting social norms, it is perhaps unsurprising that attitudes to technology have been uncorrelated with parenting app uptake [37]. The case vignettes illustrated how included parents had explored many other solutions in the past but maintained hope that the next solution would improve family life, and this is valuable in itself, in that when parents are more hopeful, there is greater parental well-being, child adjustment, and family resilience [38].

### Strengths and Limitations

This study provides novel insights into how parents use an app with in-the-moment advice for supporting children through behavioral and emotional challenges. The use of qualitative analysis captured the diversity of views and uncovered some unexpected results. The use of illustrative case summaries illustrated how the app fitted into the wider context of family life, a feature often missing from pilot studies. However, app use was all self-reported, and no objective analytics were used, and as such, there is a risk that social desirability bias could lead to upwardly biased reports. Moreover, the way parents used the app in unexpected ways would have confounded objective analytics. The sample size was appropriate to evaluate the way parents used the app and to inform future development, but it was too small to draw conclusions about the proportions of parents who used the app in different ways or to make inferences about changes in child behavior [22,23]. This study used a self-selecting sample who may be more open to parenting apps than the general population of parents seeking parenting support, potentially leading to greater reported enthusiasm for the app than would be found with a general population sample. In addition, we were unable to recruit any fathers into this study, limiting the generalizability of this study to mothers.

### Implications for Future Research

This study has implications for people developing apps to support parenting skills. Scholars should expect some resistance from parents who are wary of digital interventions but should also anticipate that other parents will nevertheless be open to using an app. Apps should facilitate several different levels of engagement (perhaps covering information, reflection, and in-the-moment guidance) that would allow parents with some qualms to begin to benefit from the app. This way, the largest number of parents can be supported by digital parenting interventions in a way they feel comfortable with. Alternatively, insights from behavioral science such as reminder notifications and incentives may be incorporated to prompt people to use parenting apps in keeping with suggestions, as has been attempted elsewhere in mental health care [39].

For a range of reasons, some parents occasionally found it impractical to use the phone in the heat of the moment. Some parents found a workaround in the use of home speakers. We suggest that future digital parenting skills programs incorporate home speakers. Although individual parenting skills have been built into home speaker apps in the past, families would benefit more from support integrating home speakers, smartwatches, and tablets with mobile apps [16].

Similarly, parenting apps should be designed to make it easy for parents to achieve their own personal goals, otherwise, parents will use the app in unintended ways that suit their family. Incorporating more personalization into parenting apps can support parents to use generic digital tools in ways that target the particular challenges affecting their families. Apps should provide evidence-based advice alongside useful tools.

Future studies should explore whether the finding that parents use digital microinterventions differently than guided is replicated among other groups of parents.

### Conclusions

This study has described how a sample of mothers used an app designed to support evidence-based parenting skills following previous attendance at a parenting program. Digital parenting support is a rapidly growing area, and it is important that a wide range of possible interventions are offered to parents so they can find an approach that suits the needs of their families. Rapid pilots, such as this study, offer a comparatively cheap way of evaluating whether an app is suitable for parents and provide an opportunity to screen for areas to improve the intervention. In the future, we recommend researchers ensure parents’ preferred approaches match up with the directions they are nudged toward by digital interventions.

## Supplementary material

10.2196/53907 Multimedia Appendix 1 Topic guides for interviews.

10.2196/53907 Multimedia Appendix 2 Case studies for Time Out Timer app.

## Abbreviations

COREQ: Consolidated Criteria for Reporting Qualitative Research
