# Supplementary material for: Evaluating a Mobile App Supporting Evidence-Based Parenting Skills: Thematic Analysis of Parent Experience
Source: JMIR Pediatr Parent. 2024 Sep 5;7:e53907. doi: 10.2196/53907 (PMC11391659; doi:10.2196/53907)
Supplement: Multimedia Appendix 1 [file pediatrics-v7-e53907-s001.docx]

**Topic Guides:**

**Interview 1**

**Part 1:**

How many children

Ages of children

Do any children have ADHD/anxiety/conduct disorder/ASD?

Do they ever use time out/naughty step/quiet corner?

What parenting skills groups/coaching/training have they had?

What parenting apps have they used?

What parenting books have they used?

Which social media do they mention they use?

Who else lives at home with the family? Eg dad/stepdad/nobody

**Part 2:**

Have you ever used time out, naughty step, or quiet corner for your child?

What word do you use for it?

How often do you tend to use it? (more than once a day, every day, most days, about once a week, about once a month, less than once a month)

What words come to mind when you think about using naughty step?

How long do you make time out last for? (in minutes)

What do you use as a timer when you do naughty step?

What other strategies do you use to manage behaviour problems?

Apart from time out, what other strategies do they use to manage behaviour?

**Part 3:**

Do you think the app would be useful?

Do you think you would find the timer function easy?

Do you think you would find the positivity and praise reminders helpful?

Do you think you would find the checklists and scrollable information helpful?

How do you think the app could be difficult to use?

How do you think the app could make life easier?

What improvements would you like to see in the app?

Would you like to download the app and try it out for 2 weeks and let us know how you get on?

**Interview 2:**

Have you used the app at all?

How many times?

Have you used the timer function?

Have you used the checklists to prepare your child?

Was the app useful or distracting?

Have you used the information pages at all?

What has been useful about the app?

What has been difficult about the app?

Is there anything you would change or improve about the app?

What did your child think about you using the app?

Would you recommend the app to a friend?

Do you expect to use the app again?
