# Supplementary material for: Evaluating a Mobile App Supporting Evidence-Based Parenting Skills: Thematic Analysis of Parent Experience
Source: JMIR Pediatr Parent. 2024 Sep 5;7:e53907. doi: 10.2196/53907 (PMC11391659; doi:10.2196/53907)
Supplement: Multimedia Appendix 2 [file pediatrics-v7-e53907-s002.docx]

**Case Studies for Time Out Timer App**

**Case study 1: Q**

Q has a large family and several of her children have neurodevelopmental disorders. She’s completed triple P in the past but feels put on the spot when asked about it; she worries she can’t remember the content. Sheepishly she mentions the distract skill. Otherwise, she has had guidance from local authority staff as she figures out how to support her children. The dynamics between her children have changed as they’ve grown up. Some of them can take themselves off to a quiet place when they start to “see red’. But more often they all fall out.

Q was interested in trying the app. She has used a book about a monster to consider calming down, but she did not consider there might be apps that could help. She was not phased by trying a digital intervention instead.

Over the following weeks she used the app with her children several times. They really enjoyed the sense check and found this helpful. But other features of the app bothered them. There were some glitches in the app where it would crash which would make everybody laugh. They would sometimes try to do the breathing exercises but they found the rate of breathing too slow and again the family ended up in hysterics together. One of her children thought the breathing was boring and they wondered whether a more engaging visual would help.

They liked the idea of using checklists to check chairs, but they thought some of the app’s information was contradictory. Altogether they found the experience “clicky and swipey”. In the end they thought the app wasn’t useful right now but suggested that with some changes it could be a useful tool for families.

**Case study 2: M**

M has three children with her ex and one with her new partner. The hardest thing is that her oldest, a ten year old son, has struggled with being co-parented. Dad sets no limits for the children and makes cruel comments about her to the children. He pushes her son too hard. When he comes home he’s angry and confused. He destroys things in the house. She says “he thinks he’s in charge of me” and attributes this to the way his dad treated her when they lived together. She sometimes worries she overlooks her other children because of the attention her son requires. She’s getting intensive one-on-one support and is interested in trying an app as an additional tool.

Initially she spent a few days reading through the whole app so she knew where everything was. She began to think more about using praise to encourage her daughter. She showed her partner the senses exercise and he used it with his own children, but changed it to dinosaurs instead of senses. She couldn’t use it with her eldest and when she started using the app with her middle son, he was really resistant. He tries to act like his brother. But over time he learned that he got out of time out quicker if he followed the rules. He even started participating with the calming activity on the time out page to blow the balloons up. She said there should be testimonials in the app saying “it works! hang in there!”

**Case study 3: S**

S was more worried about her 5 year old son who is waiting for neurodevelopmental input than about her two year old daughter. She’s had completed Triple P Stepping Stones with extra personalized input on the way through because of his behaviour. She’s also read lots of books about toileting lately and has lots of ideas. She uses time out on the bottom step with a range of egg timers which he likes looking at, but she finds it exhausting getting him over to sit down. She tries to articulate time out as ‘mum needs a break’. But she sometimes just puts the iPad on the top shelf instead. She tried printing out a sheet of rules to laminate and put in his room, but it wasn’t to hand when they needed it.

At least a mobile app would provide information whenever she needed it. As soon as she saw it she thought it would be hard to get it out in the heat of the moment. She could imagine having her hands literally full and trying to gesture towards the bottom step. The idea of the breathing exercises caught her imagination and she was interested in supporting her son with calming activities.

When she had the app she enjoyed scrolling through for advice but she never managed to get it out during a time out. In the heat of the moment she always forgot. Plus her son liked the visual stimulation of the egg timer. She had been surprised by the meme style images – it wasn’t that she didn’t like the images but they just seemed out of keeping with the app style. The information was actually quite useful and after reading them she made a point of giving her daughter more encouragement.

**Case study 4: L**

L's 7 year old daughter has a lock on her bedroom door. She has two older brothers and the oldest has ASD and emotional dysregulation, often becoming violent at home. Social services suggested giving L’s daughter a lock so she could lock herself in her room and feel safe. Anxiety has been an increasing problem for her so her mum encourages her to talk through her problems at the end of the day and to journal.

They took the app away on holiday because past experience has revealed that airports can be difficult. L’s son often uses the 5-senses technique to calm down and L’s daughter was happy to try the calming breaths. Both of them found that they could use the app on their own during the holiday to manage difficult emotions. L’s daughter could often be found on her mum’s phone taking deep breaths calming breaths. L’s son even asked for the phone a couple of times to try to calm down. They didn’t use it for “time outs” and thought the instructions around calming time were better suited for neurotypical children. As a former teacher, L didn’t need advice about using encouragement, but thought it was helpful. In her professional opinion electronic devices can be problematic, but seeing her son and daughter using the app made her rethink: they use electronics for everything else, why not for mental health too? After all, it made life much easier for her and her husband who didn’t have to carry cards explaining relaxation techniques everywhere they go.

**Case study 5: O**

O has four children aged between 5 and 18. Two have diagnoses of autism and she considers herself experienced and confident at managing behaviour problems. She uses a range of timers to manage her children’s expectations about break times and has a range of inflatable balls her children bounce on to self-regulate. Massage, running around, beauty therapy, or reading a book: she describes each child’s calming regime. She was recruited via a children’s sport group and was happy to share her opinion on the app.

When she used the app she found there were too many clicks to get between different pages. In the heat of the moment, it was difficult to set timers. She enjoyed reviewing the information pages. The breathing page was a nice idea but even that seemed to be difficult to navigate to. The idea of looking at a phone during an important parenting moment did not sit well with her; in fact she thought that was a bad idea. But she noted that younger parents – on their first child, perhaps their second - would likely find the app very useful. It just wasn’t right for her.

**Case study 6: H**

Before she had her twins - both 3 years old now - H used to work at a parenting assessment centre. She also has a 6 year old who can become violent and sometimes hits her. She finds it hard to know how to react. She’s completed Triple P in the past and he is awaiting a neurodevelopmental assessment. H was interested in trying out the app to give her new ideas and resources for managing this violence. She sometimes used time outs, but she found it difficult because their house is quite open plan so there’s not much privacy. More likely, her 6 year old announces his own time out and takes himself off to his bedroom.

She read through the information pages when she got the app and tried to start using more praise. She was surprised to see that she didn’t have to insist her children apologised when they were done and found that this change made life easier. She mentioned she had re-read the information pages before we met. In fact, she had memorised so much of the information on the app that she did not use the timer; instead she used the kitchen Alexa and the Google Home in the lounge to set the timers. The main problem with the app is that she doesn’t always have her phone on her; then again sometimes the smart speakers do not recognise her voice so no technology is perfect.

**Case study 7: W**

W has two daughters, an 8 year old and an 11 year old. She particularly worries about the eldest, who she describes as emotionally more like and 8 or 9 year old and is on the neurodevelopmental waiting list. They spend time together doing colouring, craft, and relaxation exercises and attend a group for children with autistic spectrum disorder. Over recent weeks, as the prospect of secondary school approaches, she has been more aggressive and has required some more time outs where she spend 30-60 minutes in her room until she can tolerate coming out.

As they started using the app, W found that the time out section of the phone didn’t help at all. Instead they used the relaxation modules. They enjoyed doing the calming breathing activity together. Soon they were using it without the app. They were more reliant on the app for the five senses relaxation activity because it can be hard to remember the order of the senses. I wondered if W’s daughter had been surprised to see her mum getting out an app on her phone. W admits “going everywhere for help” so her daughter probably thought “it’s just another thing mum’s been looking up”. She’s taking it in her stride.

**Case study 8: E**

E was one of the youngest participants and didn’t want to talk to me alone so she brought her friend L with her. At 2 years old, E’s son is at the bottom of the range for the app and approaches like time out wouldn’t be any help. But her supporters thought it could be useful to work on calming, distraction, and praise using the app and she was totally up for trying it. She does worry about her son’s tantrums and his language development is a little behind her friend’s daughter’s so she’s keen to keep trying new ways to support him.

When we opened the app she gasped and exclaimed “Ohh my my I actually wasn't... Not me being fascinated by a timer.” She thought it was really easy to use and was happy to give it a go. Over the coming weeks she followed the guidance to use more praise and encouragement. She tried using the time out timers soon realising that her son was the wrong age. Her 2 year old was also too young to engage with the breathing exercises. Often the best way to calm him down was singing to him and distracting him with colours. She could imagine an app with colourful characters and distracting games using the characters. She talked about simplifying the senses: what if we just asked for one of each thing? And she suggested that blowing out birthday candles might help little ones relax and focus.

**Case study 9: V**

Sometimes when your child is really winding you up and upsetting her siblings, V explains, it is hard to manage your own emotions. When everybody has fallen out, you know what they really need is a hug to make everything ok again, but you don’t really feel like hugging them. It’s good to have the app as a reminder to do that.

V has four children. It’s not that they’re naughty children but they do get really upset if she buys the wrong things from the shop. She tries to take their PS5 off them when they are badly behaved. She doesn’t smack her children but she thinks some people probably do and reckons that the app creates an alternative model for their relationship, even suggesting that ending with a hug will have positive oxytocin effects on family life.

She used the app 2-3 times each week, including half term, but didn’t need it the week before we spoke. Her son got the hang of taking 30 seconds of calming down, but her daughter was a little confused about what she was supposed to do. By the end of the three weeks her son knew what was going to happen when she took her phone out and could anticipate calming time.

**Case study 10: N**

“It’s like us having a cup of tea”, N tells me, “they’re like a completely different person after”. N is weaning her daughter after long-term breastfeeding. Now she is unable to use milk to support emotional regulation, she is looking for other approaches to managing behaviour problems. She has found that a more direct and ‘authoritarian’ approach is effective with some children but not others. She finds it useful to use consequences but when she used time out her son picked up the “thinking chair” and smashed it against the wall.

When N started using the app she found her youngest really enjoyed using the breathing. They even incorporated it into her bedtime routine. Her mum notes that some children struggle with looking at a phone before bed, but it doesn’t bother her daughter. Sometimes to calm down they just sit next to each other and take the deep breathes for 30 seconds, but sometimes her daughter wants to keep going so they set the timer for the maximum 120 seconds. Her daughter still gets angry sometimes and becomes physically violent with siblings but when she does she has learned to try to avoid time out. She tries to negotiate, “No, I don't want time out. Can I just do breathing?" N is wise to this ploy, but quietly pleased that her daughter is enjoying the calming breathes they do together.
